# Oxygen insufflation via the working channel during tracheal intubation guided by a flexible optical scope and benefits, dangers, and future of the method: a narrative review

**DOI:** 10.1016/j.bjao.2024.100346

**Published:** 2024-10-17

**Authors:** Alexandre Garioud, Michael Seltz Kristensen

**Affiliations:** Department of Anaesthesia, Centre of Head and Orthopaedics, Rigshospitalet, University Hospital of Copenhagen, Copenhagen, Denmark

**Keywords:** Anticipated difficult airway management, Flexible optical, Intubation, Oxygen insufflation, Working channel

## Abstract

When performing tracheal intubation guided by a flexible optical scope, the operator may lose the anatomical orientation and hypoxia may occur. Oxygen insufflation through the working channel of the flexible optical scope is used by anaesthetists to prevent blurring of the lens and to prevent hypoxia. However, fatal iatrogenic injuries from this method are reported. Our aim is to review the current literature on oxygen insufflation through the working channel during tracheal intubation guided by a flexible optical scope, to identify its benefits and the source of its dangers, and ultimately to provide a basis for the development of a safer technique. We conducted a literature search of databases, guidelines, and textbooks using search terms related to oxygen insufflation through the working channel during intubation guided by a flexible optical scope. Clinical trials confirm that the technique results in better visibility and better oxygenation during intubation. Gastric rupture and pneumothorax were the most frequent types of injury. We identified that oxygen insufflation without pressure limitation during accidental misplacement of the flexible optical scope in the oesophagus, deep in the lower airways, or via a tear of the airway mucosa was the cause of barotrauma. We conclude that a delivered pressure below 40 cm H_2_O will likely carry a low risk of serious adverse outcomes. The technique in its current form seems unsafe, and future research should aim at developing a system that delivers oxygen at pressures respecting gastric and airway physiologic pressure thresholds with a flow sufficient to obtain the documented advantages of the technique.

Tracheal intubation guided by a flexible optical scope, often still called ‘fibreoptic intubation’, remains an indispensable technique for safely managing the anticipated difficult airway.^1^ Nevertheless, acquiring and maintaining proficiency with the technique is proving progressively challenging with relatively few opportunities to apply and practise it.^1^ Therefore, it is imperative to explore and integrate methodologies that could enhance the ease and safety of intubation guided by a flexible optical scope.

Obstruction of the lens during intubation guided by a flexible optical scope by secretions, haemorrhage, or vomit leads to loss of anatomical orientation and combined with oxygen desaturation can give rise to major challenges during the procedure leading to higher rates of unsuccessful intubation.^2^ Nasal high-flow oxygenation, antisialogogues, and suction through the working channel of the flexible optical scope are useful adjuncts to circumvent such challenges but may be insufficient to optimise intubating conditions.^2^ In the 1980s, airway management specialists started to recommend oxygen insufflation through the working channel of the flexible optical scope.^3^, ^4^, ^5^ This method is in widespread use today.^6^, ^7^, ^8^, ^9^ A constant flow of oxygen through the distal tip of the flexible optical scope clears the lens, prevents accumulation of liquids, and can help maintain normoxaemia.^5^^,^^10^ Despite the success of this procedure, there have been reports of fatal iatrogenic injuries during intubation using this method, thereby questioning the safety of the technique.^11^, ^12^, ^13^, ^14^ We aimed to review the current literature on oxygen insufflation through the working channel during intubation guided by a flexible optical scope, to identify its benefits and the source of its dangers, and ultimately to provide a basis for the development of a safer technique.

## Search methodology

This narrative review follows the 2020 PRISMA guidelines.^15^ The PubMed, Cochrane Central, Embase, Scopus, and Cinahl databases were searched in September 2023, without filters, using keywords: intubation, intratracheal [Mesh] or intubation, fibreoptic and oxygen insufflation or oxygenation or oxygen supplementation. Major airway management textbooks and UpToDate (https://www.wolterskluwer.com/en/solutions/uptodate) articles were scrutinised.^2^^,^^16^, ^17^, ^18^, ^19^, ^20^, ^21^ Title and abstract screening, study inclusion, and data extraction were done by one author (AG) using the software Covidence™ (www.covidence.org). Article reference lists were subsequently manually screened for any additional relevant articles. Inclusion criteria were any article studying and reporting the effects of tracheal intubation guided by a flexible optical scope using oxygen insufflation through the working or suction channel of the flexible optical scope during the intubation. Exclusion criteria were articles describing the method only or articles describing the use of other oxygenation techniques or oxygen insufflation in patients whose trachea was already intubated. Literature type, equipment used, the applied flow/pressure of oxygen, and reported benefits or adverse effects of the method were extracted to a table.

## Summary of findings

**Fig 1:**
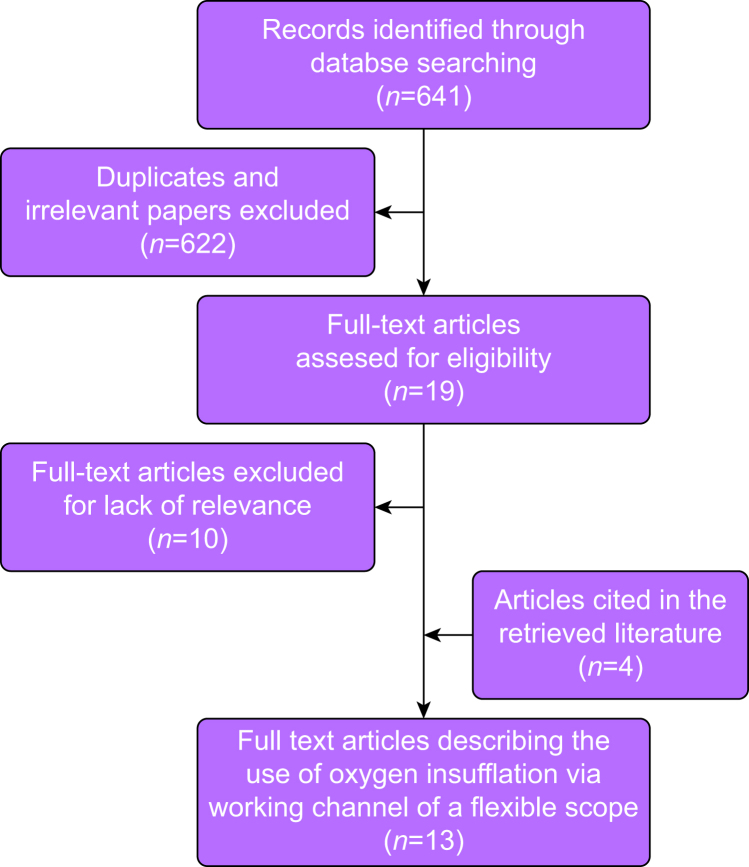
Study selection flowchart.

The literature search is presented in Fig. 1. We screened 641 articles, 622 were deemed irrelevant from title and abstract review, and 19 were retrieved for full-text review of which nine were finally included for data extraction. Manual screening of the reference lists of included articles added four articles for a total of 13 included articles for data extraction. The extracted data are presented in Table 1.

**Table 1:** Extracted data from reviewed literature in chronological order.

Publication	Year	Literature type	Flexible scope model	Flow	Pressure limit	Benefits	Adverse events
Rothenberg and colleagues^4^	1989	Letter to editor	Pentax Model FB-I.SH	3–4 L min^−1^ continuous	None reported	Superior oxygenation and secretion clearance	None reported
Hershey and Hannenberg^11^	1996	Case report	Pentax FI-10P	3 L min^−1^	None reported	None reported	Gastric rupture, pneumoperitoneum, gastric haemorrhage
Richardson and Dooley^12^	1996	Case report	Olympus LF-2	5 L min^−1^ intermittent	3500 cm H_2_O	None reported	Posterior nasopharyngeal wall puncture and facial, cervical, thoracic subcutaneous emphysema. Death after 21 days
Roberts^24^	1991	Special article	Olympus LF-1	7 L min^−1^ continuous	None reported	Defogging lens, secretion/blood clearance	None reported
Heidegger and colleagues^23^	2003	Prospective descriptive study	Olympus LF-2	4 L min^−1^ continuous	None reported	None reported	None reported
Ho and colleagues^13^	2005	Case report	Pentax FI-10RBS	5 L min^−1^	None reported	None reported	Gastric rupture, pneumoperitoneum, death owing to complications
Chapman^25^	2008	Case report	Olympus BF-P160	5 L min^−1^	None reported	None reported	Gastric rupture, pneumoperitoneum, death owing to complications
Piepho and colleagues^22^	2009	Comparative study	Karl Storz GmbH & Co.	3 L min^−1^ continuous	None reported	Superior oxygenation and local anaesthetic topicalisation, shorter intubation intervals	None observed
Khan and colleagues^14^	2010	Case report	Olympus LF-DP	2 L min^−1^	None reported	None reported	Tension pneumothorax, pneumopericardium
Lee and colleagues^6^	2018	Case report	Olympus LF-V	5 L min^−1^	None reported	Insufflation superior to suction for clearing lens, less desaturation	None observed
Roh and colleagues^7^	2020	Prospective randomised controlled study	Karl Stortz GmbH & CO	5 L min^−1^ continuous	None reported	Significantly less *P*a_O2_ decrease (*p*<0.012) than control group and reduced velocity of *P*a_O2_ decrease (*p*<0.001)	None observed
Cruz and Simon^8^	2022	Case report	Karl Storz 3.7×65	5 L min^−1^	None reported	None reported	Pneumothorax, cardiac arrest. Paediatric case
Rajan and colleagues^9^	2022	Prospective randomised study	Karl Storz 11301 BN1	6 L min^−1^ intermittent	None reported	Less need for suction to clear view. Significantly less desaturation and need of supplemental oxygen *P*<0.002	None observed

We included seven case reports, four clinical studies, one special article, and one letter to the editor. The type of equipment type varied: five articles reported use of scope models from Olympus™, four from Karl Storz, and three from Pentax. The oxygen flow rate through the working channel of the flexible optical scope varied from 2 to 7 L min^−1^. One article only reported the pressure at which oxygen was delivered to the flow meter (50 psi≈3500 cm H_2_O).^12^ However, all articles implied that oxygen was delivered either from the anaesthesia machine flow meter or directly from the flow meter connected to the hospital oxygen delivery pipeline network. Although the insufflation oxygen through the working channel of a flexible optical scope was reported to clear liquids and fogging simultaneously and to provide superior oxygenation 35 yr ago,^3^ literature on this topic is sparse. The majority of the literature describing the technique or reporting its effects was published in the 1990s. The technique was revisited by the scientific community in the late 2010s and 2020s when several clinical studies and case reports were published.^6^, ^7^, ^8^, ^9^ The potential detrimental effects have led some authors to publish recommendations against the use of the technique.^21^

Benefits from insufflating oxygen via the working channel during intubation guided by a flexible optical scope.

Six of the included articles reported benefits from the method. The comparative clinical trials all reported significantly less oxygen desaturation when compared with conventional oxygenation techniques.^7^^,^^9^^,^^22^ Additionally, one or more of the following benefits were reported in the comparative studies: shorter intubation time, less need for suction to clear the view, or better vaporisation of local anaesthetics.^7^^,^^9^^,^^22^ There were no reported differences in successful intubation rates or ease of intubation.^7^^,^^9^^,^^22^ In case reports or expert opinion statements, better clearance of secretion/blood, defogging of the lens, and better oxygenation are the major reported benefits.^4^^,^^6^^,^^24^

Dangers and adverse effects reported from insufflating oxygen via the working channel during intubation guided by a flexible optical scope.

We identified six cases of serious adverse events.^8^^,^^11^, ^12^, ^13^, ^14^^,^^25^ Gastric rupture^11^^,^^13^^,^^25^ leading to pneumoperitoneum is the most frequently reported complication (*n*=3). Other complications included pneumothorax,^8^ tension pneumothorax,^14^ posterior nasopharyngeal wall puncture leading to facial, cervical, or thoracic subcutaneous emphysema,^12^ or pneumopericardium.^14^

The mechanism for the adverse events seems to be that when intubation guided by a flexible optical scope is difficult and anatomical landmarks are not recognised, the unintentional placement of the tip of the scope either in the oesophagus, deep in the lower airways, or via a tear of the mucosa lining the airway during simultaneous oxygen insufflation resulted in oxygen entrapment, overdistention, and barotrauma of fragile organs. This resulted in the rupture of the stomach,^11^^,^^13^^,^^25^ a bronchus, bronchioles, or alveoli.^8 14^ One case reported involuntary perforation of the pharyngeal wall with subsequent insufflation in the surrounding soft tissues resulting in facial, cervical, and thoracic subcutaneous emphysema.^12^ In three of the six cases, death occurred as a direct or indirect consequence of the complication.^12^^,^^13^^,^^25^

The *incidence* of complications is difficult to estimate as there were no reports of adverse events in the comparative studies^7^^,^^9^^,^^22^ that included 34, 36, and 40 patients, respectively. The comparative studies excluded patients with an anticipated difficult airway or patients with pre-existing pulmonary diseases,^7^ with known airway pathology (nasal tumours, lesions on the posterior tongue, pharyngeal, or laryngeal areas) along the pathway of insertion of the flexible optical scope^9^ or patients with ASA classification greater than 3. In two of the comparative studies, intubation guided by a flexible optical scope was performed only after induction of general anaesthesia.^7^^,^^22^ All three studies exclusively included patients undergoing elective surgery.^22^ It can thus be argued that the patients included in the comparative studies are highly selected for optimal conditions for intubation guided by a flexible optical scope and thus had a relatively low risk of scope misplacement and possible subsequent injuries and that these studies contribute little to the determination of the safety of the technique.^3^

In a study of 1600 cases relying on a standardised protocol in a unit with a high frequency of intubation guided by a flexible optical scope, oxygen flow rates of 4 L min^−1^ were insufflated through the working channel of the flexible optical scope with a high success rate of intubation and without reported injuries.^23^ This suggests that adverse events can be minimised, but when they occur are serious and potentially fatal.

All studies reported the oxygen flow used during the intubation guided by a flexible optical scope and that oxygen was delivered either from the anaesthesia machine flow meter or directly from the flow meter connected to the hospital oxygen delivery pipeline network. However, the case report by Richardson and Dooley^12^ is the only article explicitly reporting the pressure at which oxygen was delivered (50 psi≈3500 cm H_2_O). The driving pressure is probably the most important factor related to the occurrence of adverse events. In general, in the UK and Europe, hospital pipeline networks deliver oxygen at pressures of 4–5 bar (58–73 psi), approximately 4100–5100 cm H_2_O.^26^

Evidence differs regarding the pressure required to perforate the gastric wall. Cadaveric studies report pressures of 50–100 mm Hg (68–136 cm H_2_O), whereas studies conducted during cardiopulmonary resuscitation report 120–150 mmHg (163–204 cm H_2_O) to be required,^27^^,^^28^ pressures ranging from 68 to 204 cm H_2_O. This corresponds to an intragastric volume of 4 L.^28^ Several studies have measured the pressures required to cause gastric insufflation during facemask ventilation in anaesthetised patients. In 2022, Gamal and colleagues^29^ reported pressures as low as 10 cm H_2_O in anaesthetised, non-paralysed patients. Other studies reported higher pressures (15–25 cm H_2_O) being sufficient to open the lower oesophageal sphincter and force oxygen into the stomach.^30^^,^^31^ In awake patients, manometry of the upper oesophageal sphincter reports resting pressures ranging 100–150 mm Hg (136–204 cm H_2_O) but decreasing to below 15 mm Hg (20 cm H_2_O) during relaxation (i.e. deglutition).^32^^,^^33^ The resting lower oesophageal sphincter pressure ranges from 15 to 30 mm Hg (20–40 cm H_2_O) and decreases to a resting gastric pressure of 3 mm Hg (4 cm H_2_O) during relaxation.^34^

An international consensus on safe ventilatory pressures recommends peak inspiratory pressure below 40 cm H_2_O and plateau pressure below 30 cm H_2_O to prevent barotrauma and volume trauma in the respiratory system.^35^

When performing intubation guided by a flexible optical scope, there is no risk of barotrauma from insufflation of oxygen, as long as the mouth and nose remain open and patent, and the tip of the scope is in a cavity (mouth, nose, or oropharynx) with an unobstructed connection with these orifices. However, during intubation guided by a flexible optical scope, one may lose anatomical orientation because of pathology, secretions, or blood. In these situations, the tip could be impacted in the oesophagus, in a mucosal cleavage, or in the peripheral distal airways. From the literature, we conclude that the driving pressures applied during oxygen insufflation through the working channel during intubation guided by a flexible optical scope exceed safe insufflation pressures and is the leading cause of the reported complications and fatalities. Oxygen delivery systems as reported in the included studies and case reports—from central delivery networks or oxygen cylinders via flow meters without pressure limitation—will deliver more than 100 times the safe pressure before insufflation stops. Therefore, we do not recommend using an oxygen source directly connected to central delivery or cylinders during oxygen insufflation via a flexible optical scope working channel, even if the system is supplied with a flow meter. In Table 2, we have summarised the probability and risks of gastric insufflation, gastric rupture, and airway barotrauma at various upper airway pressures based on the abovementioned gastric and respiratory physiological measurements and recommendations. An insufflation pressure ≤10 cm H_2_O will pose a negligible risk. Pressures between 10 and 30 cm H_2_O during a brief time frame may cause minor gastric insufflation but are unlikely to harm the awake fasting patient. Pressures between 30 and 40 cm H_2_O pose a high probability for gastric insufflation but a low risk of barotrauma and a negligible risk of gastric rupture and could be justified for a brief period if the benefit of clearing the lens and improving oxygenation clearly outweighs this risk.

**Table 2:** Probability/risk of gastric insufflation, gastric rupture, and airway barotrauma at various upper airway pressures, estimations derived from the literature. ∗Cadaver data.

Pressure (cm H_2_O)	Probability of gastric insufflation	Risk of gastric rupture^26^^,^^27^	Risk of airway barotrauma/pneumothorax
<10	Negligible^29^	Negligible	Negligible
10–15	Low^30^^,^^31^	Negligible	Negligible^35^
15–29	High^30^^,^^31^^,^^34^	Negligible	Negligible^35^
30–40	High	Negligible	Low^35^
40–67	High	Negligible	High^35^
68∗–163	High	Possible	High
>163	High	High	High

Intermittent oxygen insufflation via the suction valve of the scope to the working channel instead of directly via the working channel is a technique utilised by some investigators.^9^^,^^12^ When using this method, oxygen flow is only present when the valve is activated thereby decreasing the delivery of oxygen compared with the conventional technique and suction cannot be applied when the suction channel is used for oxygen insufflation. Additionally, intermittent instead of constant oxygen flow does not guarantee the avoidance of serious complications as demonstrated in the case from Richardson and Dooley^12^ (Table 1).

The application of high-flow nasal oxygenation offers a widely accepted alternative for efficiently improving oxygenation during awake intubation with a flexible optical scope.^36^ However, high-flow nasal oxygenation may not always be available even in a first-world setting and the high costs are significant limitations for its use in budgetary-constrained, poorly resourced environments.^37^ Furthermore, to our knowledge, no clearance of the lens of the flexible optical scope from secretions has been documented from the addition of high-flow nasal oxygenation during awake tracheal intubation.

We believe that a method having the ability to clear and keep liquids away from the lens of a flexible optical scope and which delivers oxygen simultaneously can be advantageous in situations where marginal gains are key to successful difficult airway management. Therefore, future research should aim at developing a system which is capable of delivering oxygen at pressures at or below 40 cm H_2_O that will likely carry a low risk of serious adverse effects. This will require an *in vitro* study to test the safety and efficacy of the system, followed by studies to test the technique in a clinical trial setting.

## Conclusions

Oxygen insufflation through the working channel during tracheal intubation guided by a flexible optical scope improves visualisation through the lens and maintains normoxaemia during this challenging procedure. Current practice using hospital pipe networks or cylinders connected via a flow meter as a source of oxygen is an unsafe method. A safer alternative delivering oxygen at pressures respecting gastric and airway physiological pressure thresholds could be a potential solution and should be investigated.

## Authors’ contributions

Conceptualisation: both authors

Data curation, formal analysis, and investigation: AG

Supervision: MSK

Methodology: MSK

Writing original draft: AG

Draft revision: both authors

Visualisation: AG

Writing review and editing: both authors

## Declaration of interest

The authors declare that they have no conflict of interest.
